# Systematic analysis of gene expression profiles reveals prognostic stratification and underlying mechanisms for muscle-invasive bladder cancer

**DOI:** 10.1186/s12935-019-1056-y

**Published:** 2019-12-16

**Authors:** Ping-Bao Zhang, Zi-Li Huang, Yong-Hua Xu, Jin Huang, Xin-Yu Huang, Xiu-Yan Huang

**Affiliations:** 1 Department of General Surgery, Shanghai Jiaotong University Affiliated Sixth People’s Hospital, 600 Yi Shan Road, Shanghai, 200233 People’s Republic of China; 2 Department of Urinary Surgery, Affiliated Hospital of Nantong University, Nantong, 226021 People’s Republic of China; 3 Department of Radiology, Xuhui Central Hospital of Zhongshan Hospital, Fudan University, Shanghai, 200031 People’s Republic of China; 4 Department of Pathology, Shanghai Jiaotong University Affiliated Sixth People’s Hospital, Shanghai, 200233 People’s Republic of China

**Keywords:** Muscle-invasive bladder cancer (MIBC), Stratification, Overall survival, Drug-target, M2 macrophage

## Abstract

**Background:**

Muscle-invasive bladder cancer (MIBC) is originated in the muscle wall of the bladder, and is the ninth most common malignancy worldwide. However, there are no reliable, accurate and robust gene signatures for MIBC prognosis prediction, which is of the importance in assisting oncologists to make a more accurate evaluation in clinical practice.

**Methods:**

This study used univariable and multivariable Cox regression models to select gene signatures and build risk prediction model, respectively. The t-test and fold change methods were used to perform the differential expression analysis. The hypergeometric test was used to test the enrichment of the differentially expressed genes in GO terms or KEGG pathways.

**Results:**

In the present study, we identified three prognostic genes, *KLK6*, *TNS1*, and *TRIM56*, as the best subset of genes for muscle-invasive bladder cancer (MIBC) risk prediction. The validation of this stratification method on two datasets demonstrated that the stratified patients exhibited significant difference in overall survival, and our stratification was superior to three other stratifications. Consistently, the high-risk group exhibited worse prognosis than low-risk group in samples with and without lymph node metastasis, distant metastasis, and radiation treatment. Moreover, the upregulated genes in high-risk MIBC were significantly enriched in several cancer-related pathways. Notably, *PDGFRB*, a receptor for platelet-derived growth factor of PI3K-Akt signaling pathway, and *TUBA1A* were identified as two targets of multiple drugs. In addition, the angiogenesis-related genes, as well as two marker genes of M2 macrophage, *CD163* and *MRC1*, were highly upregulated in high-risk MIBC.

**Conclusions:**

In summary, this study investigated the underlying molecular mechanism and potential therapeutic targets associated with worse prognosis of high-risk MIBC, which could improve our understanding of progression of MIBC and provide new therapeutic strategies for the MIBC patients.

## Background

Muscle-invasive bladder cancer (MIBC) refers to cancers happening in the muscle wall of the bladder. Symptoms such as pain with urination, blood in the urine, and low back pain are often observed in patients with bladder cancer. Bladder cancer is one of the most common malignancies worldwide [1]. It is much more commonly diagnosed in men than in women, but female patients are usually with more advanced stages at the time of diagnosis, and exhibit less favorable survival [2]. MIBC has the potential to spread to nearby lymph nodes and other organs. In severe cases, metastasis would affect distant organs such as lungs and liver [3]. Increasing age is considered to be the main risk factor for bladder cancer, and impacts from smoking and exposure to some industrial chemicals are also reported to be significant [4].

With the advances in high-throughput technologies, several prognostic biomarkers have been revealed previously. Genetically, genome-wide association studies (GWAS) have revealed that genes on chromosome 8q24, particularly the *PSCA* gene (Prostate Stem Cell Antigen), were associated with increased metastatic potential of bladder cancer [5, 6]. A hypothesis reasons that these genes detected by GWAS may be associated with androgen receptor responsiveness and inducing androgen-independent pathways, which stimulates tumor growth [5]. The losses of regions on 10q (including *PTEN*), 16q, and 22q, and gains on 10p, 11q, 12p, 19p, and 19q were positively associated with metastasis in muscle-invasive bladder cancers [7]. With the genome-wide gene expression data, several studies have identified a combination of gene signatures to predict the prognosis of MIBC. Specifically, four gene signatures, *IL1B*, *S100A8*, *S100A9* and *EGFR*, have been reported to have the capability of predicting MIBC progression [8]. The novel combination markers of *USP18* and *DGCR2* can also predict survival in patients with muscle invasive bladder cancer [9]. In addition, *NR1H3* expression is identified as a prognostic factor of overall survival for patients with muscle-invasive bladder cancer [10]. However, there are some limitations for these studies. First, the gene signatures identified by these studies were not robust due to lack of validation dataset or small sample size in validation dataset. Second, comparative analysis was not conducted on the performance of these gene signatures for MIBC prognostic prediction. Third, the potential mechanism resulting in the worse prognosis has not been thoroughly investigated. In addition, the potential therapeutics for patients with worse prognosis was not proposed by these studies. In the present study, to avoid these limitations, we attempted to detect a combination of gene signatures for MIBC prognostic prediction and stratification. Based on the prognostic stratification, we also investigated the underlying molecular mechanism and potential therapeutic targets associated with worse prognosis of high-risk MIBC, which could improve our understanding of MIBC progression and provide new therapeutic approaches for these high-risk patients.

## Materials and methods

### Data collection and pre-processing

The TCGA-BLCA gene expression datasets [11] and corresponding clinical data were obtained from UCSC Xena Browser [12] (https://xenabrowser.net/datapages/). The E-MTAB-1803 dataset [13] was downloaded from ArrayExpress (http://www.ebi.ac.uk/arrayexpress/) database [14]. The TCGA-BLCA dataset was divided into two subsets for model training and validation, using random sampling without replacement. For each gene in the three datasets, the expression values were discretized as high or low expression if the expression values higher or lower than its corresponding median.

### Gene expression data of MIBC cell lines

We also collected the normalized gene expression data of 30 MIBC cell lines from Gene Expression Omnibus (GEO) database [15], with accession number GSE47992 [16]. The Wilcoxon rank-sum test and fold change method were used to identify differentially expressed genes between two conditions.

### Overrepresentation enrichment analysis (ORA)

Overrepresentation enrichment analysis, which was based on hypergeometric test, was implemented by R package *clusterProfiler* with *enrichKEGG* function [17]. We chose adjusted P-value 0.05 as the threshold for the selection of significant pathways.

### Gene set enrichment analysis

The gene set enrichment analysis was implemented in R/Bioconductor fgsea [18]. The genes were pre-ranked based on the Z statistic obtained in a differential expression analysis between high-risk and low-risk groups. 1000 permutations were used to calculate the enrichment significance.

### Cox proportional hazards regression analysis

Cox proportional hazards regression analysis was performed to evaluate the differences in overall survival between patients from two risk groups or two expression status, which was implemented using R package *survival* with *coxph* function. Kaplan–Meier curves were plotted to visualize the overall survival of each group. The risk score for each patient was calculated based on the expression of three gene signatures selected by *predict.coxph* function. These three signature genes were selected from previously identified prognostic gene pool by Maximum Minimum Parents and Children (MMPC) algorithm [19], which was implemented by R package *MXM* with MMPC function.

### Drug-target analysis

The drug-target analysis aimed to explore drugs that are capable of inhibiting thos upregulated genes in high-risk MIBC. The drug–target interactions were extracted from Drug Gene Interaction Database [20] (DGIdb) using the R package *maftools* with *drugInteractions* function [21]. These interactions were visualized by Cytoscape 3.7.1 [22].

### Statistical analysis

R version 3.6.0 was used to perform all analyses. Statistical comparisons between groups were performed using the t-test or non-parametric Wilcoxon rank-sum test. P < 0.05 was considered as indicative of statistically significant differences.

## Results

### Identification of prognostic genes and construction of prognostic model for MIBC

**Fig. 1:**
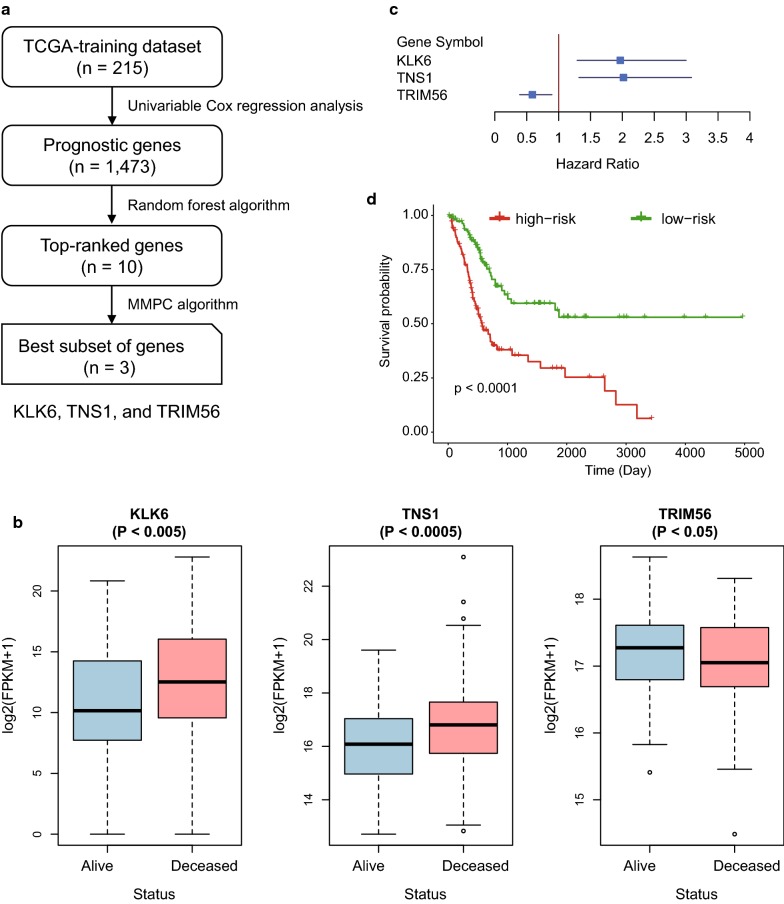
The development and construction of Cox proportional hazard regression model of three-gene-signature. **a** The workflow for the identification of three prognostic gene signatures. **b** The expression levels of the three gene signatures in alive and deceased patients. **c** The hazard ratio and 95% confidence interval of the three gene signatures in the Cox proportional hazard regression model. **d** The Kaplan–Meier curves for the two risk groups in the TCGA-training dataset. The red and green curves represent the high-risk and low-risk groups, respectively

To select prognostic genes for prognostic model construction, we designed a systematic data analysis workflow to search for a subset of genes. We first divided the samples from TCGA into training and validation datasets, which were labeled as TCGA-training (n = 215) and TCGA-validation (n = 214), respectively. Univariable Cox proportional hazards regression analysis was conducted to identify a total of 1473 prognostic genes (Log-rank test, *P* < 0.05). These prognostic genes were then ranked by their importance estimated by random forest algorithm. The univariable Cox regression analysis of the top-ten genes were listed in Table 1. Subsequently, the Maximum Minimum Parents and Children (MMPC) algorithm successfully identified three prognostic genes, *KLK6*, *TNS1*, and *TRIM56*, as the best subset of genes (threshold for MMPC = 0.05). As shown in Fig. 1b, c, *KLK6* and TNS1 were more abundantly expressed in deceased patients than in living patients (Wilcoxon rank-sum test, *P* < 0.005), and their expression patterns were negatively correlated with patients’ overall survival, while higher expression of *TRIM56* was observed in living patients (Wilcoxon rank-sum test, *P* < 0.05), indicating a favorable prognosis. Finally, multivariable Cox proportional hazards regression model was constructed based on these three prognostic genes, and the patients were divided into high-risk and low-risk groups based on their risk scores estimated by the Cox model (median of the risk score as the threshold). As illustrated in Fig. 1d, the samples from high-risk and low-risk groups exhibited significantly different prognostic outcomes (Log-rank test, *P*-value < 0.0001), suggesting that the stratification by the Cox model was closely associated with MIBC overall survival.

**Table 1:** The top-ten prognostic genes ranked by random-forest-based importance

Gene symbol	coef	exp(coef)	se(coef)	Z-score	Pr(> |Z|)
KLK6	0.67	1.96	0.21	3.12	1.80E−03
RGMA	0.64	1.89	0.21	2.97	3.00E−03
TNS1	0.63	1.87	0.22	2.90	3.69E–03
P4HA3	0.54	1.72	0.21	2.55	1.07E–02
UACA	0.50	1.65	0.21	2.35	1.87E–02
CYTL1	0.47	1.60	0.21	2.21	2.71E–02
PTCD3	− 0.45	0.64	0.21	− 2.12	3.40E–02
TRIM56	− 0.43	0.65	0.21	− 2.03	4.27E–02
NRP1	0.42	1.52	0.21	1.97	4.92E–02
NHS	− 0.42	0.66	0.21	− 1.97	4.93E–02

To investigate the biological function of the three prognostic genes in MIBC, we collected 30 MIBC cell lines. For each of the three prognostic genes, we calculated their expression in each cell line, ranked them and selected the first four as cell lines with the high expression and the last four as ones with the low expression, respectively. We then compared these four highest expression cell lines with the corresponding four lowest expression cell lines. Subsequently, KEGG enrichment analysis revealed that differentially expressed genes (DEGs) in *KLK6* high expression cell lines were enriched in pathways such as tight junction and cell adhesion molecules (*P* < 0.05, Additional file 1: Table S1), suggesting that high expression of *KLK6* in MIBC may be associated worse prognosis via regulation of cell–cell communication. Moreover, KEGG analysis of DEGs between cells with high and low expression of *TRIM56* revealed that *TRIM56* was highly associated with mismatch repair (MMR). Low expression of *TRIM56* in MIBC may be associated with the defect in MMR (Additional file 1: Table S1). In addition, we did not observe any KEGG pathways enriched by the DEGs related to *TNS1*. However, previous studies [23, 24] have reported that *TNS1* could increase the metastatic potential and alter expression of genes involved in cell motility in colorectal cancer, and may be a potential prognostic biomarker in human colorectal cancer. These results indicated that *KLK6* and *TRIM56* may be associated with worse prognosis of MIBC via regulating cell–cell communication and MMR, respectively.

### Validation of the prognostic stratification in two datasets

**Fig. 2:**
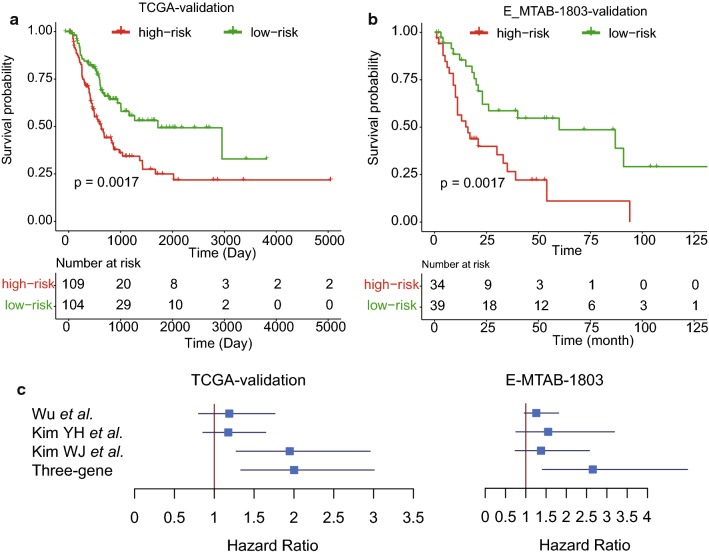
The performance of the stratification based on the three-gene-signature in two validation datasets. The Kaplan–Meier curves for the two risk groups in the TCGA-training and E-MTAB-1803 datasets were illustrated in **a**, **b**. **c** The comparison of our stratification with another three stratifications in the two validation datasets

To validate our prognostic stratification in MIBC risk prediction, we first preformed stratification on the samples (n = 214) from TCGA-validation dataset. Moreover, we also collected another gene expression dataset E-MTAB-1803 with detailed follow-up information from the ArrayExpress database. A total of 73 samples with follow-up information was included for stratification (see “Materials and methods”). Remarkably, the stratified groups in each of the two validation datasets exhibited significant difference in overall survival (Fig. 2, *P* < 0.005), suggesting that the prognostic stratification for MIBC was robust.

To further demonstrate the performance of the three-gene-signature-based stratification in MIBC cohorts, we compared our method with three other stratification methods based on the three-gene-signature with three other stratification methods proposed by Wu et al. [10], Kim et al. [8, 9], which were used to predict the overall survival of MIBC. We found that our stratification based on three-gene-signature was superior to the others (Fig. 2c). Although the stratification by Wu et al. showed similar performance with ours in TCGA-validation dataset, its performance on E-MTAB-1803 dataset was much poorer than the stratification by three-gene-signature. These results suggested that our stratification exhibited better performance in predicting overall survival for MIBC.

### The stratification was a prognostic factor independent of clinically prognostic indicators and radiation treatment

As our prognostic model exhibited satisfying performance on all MIBC patients from both training and validation sets, it was also necessary to investigate whether this stratification was a prognostic factor independent of clinically prognostic indicators, such as lymph node and distant metastasis, and radiation treatment. We constructed a multivariable Cox regression model using the three gene signatures and other clinical cofactors-such as lymph node, distant metastasis, and radiation treatment-as variables, and observed that these three genes were significantly associated with the prognosis in Cox models with and without these clinical cofactors (Table 2), suggesting that the three gene signatures still maintained prognostic significance in the multivariable regression model with the clinical factors.

**Table 2:** The multivariable Cox models with and without clinical factors including lymph node, distant metastasis, and radiation treatment

	Cox model without clinical cofactors					Cox model without clinical cofactors				
	coef	exp(coef)	se(coef)	z	Pr(> |Z|)	coef	exp(coef)	se(coef)	z	Pr(> |Z|)
KLK6	0.6767	1.9673	0.2135	3.1440	0.0017	0.6910	1.9957	0.2199	3.1425	0.0017
TNS1	0.7012	2.0161	0.2171	3.2300	0.0012	0.5928	1.8091	0.2250	2.6354	0.0084
TRIM56	− 0.5287	0.5894	0.2135	− 2.4760	0.0133	− 0.5554	0.5738	0.2180	− 2.5474	0.0109
Lymph node (yes)	–	–	–	–	–	0.5065	1.6595	0.2220	2.2817	0.0225
Distant metastasis (yes)	–	–	–	–	–	0.6021	1.8259	0.2209	2.7260	0.0064
Radiation (yes)	–	–	–	–	–	0.7972	2.2194	0.3664	2.1757	0.0296

coef: coefficients for the variables included in Cox model; Pr(> |Z|): P-value for the variables

**Fig. 3:**
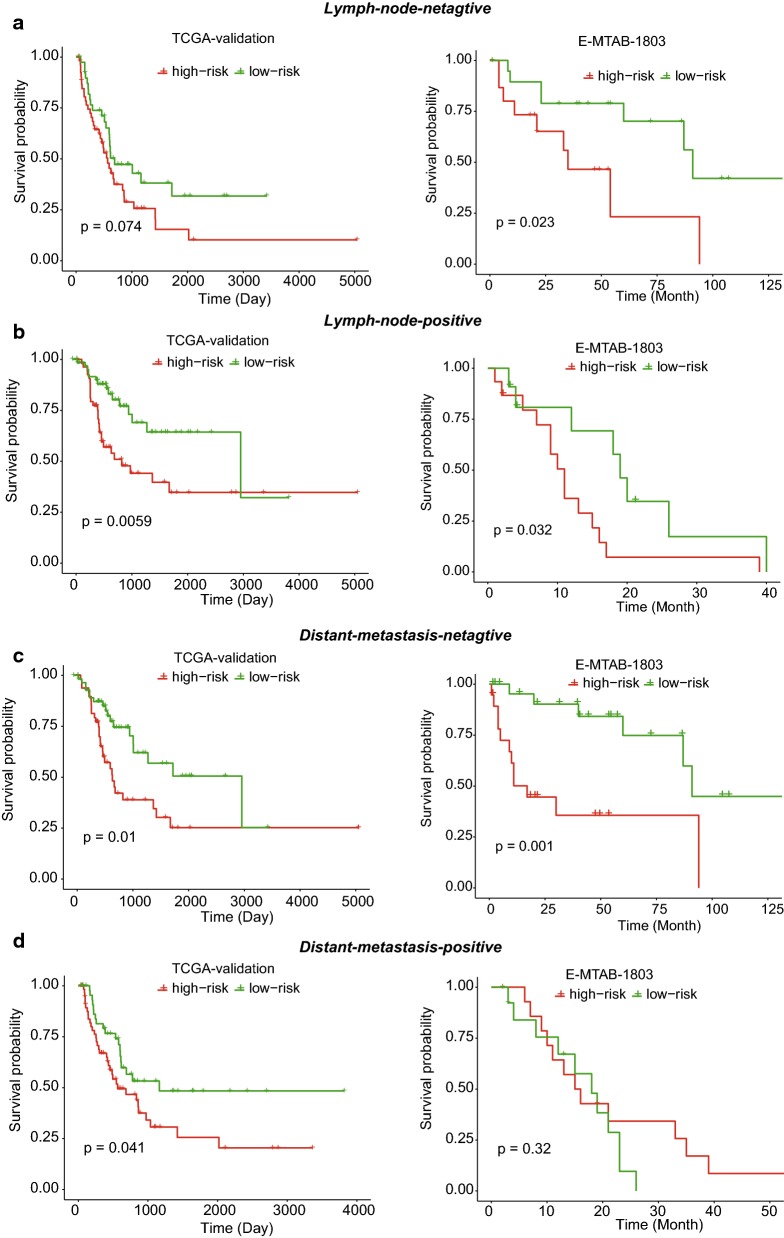
The independence of our stratification on the clinically prognostic symptoms. The KM-curves for samples with and without lymph node metastasis and distant metastasis were displayed in **b**, **a**, **d**, and **c**

**Fig. 4:**
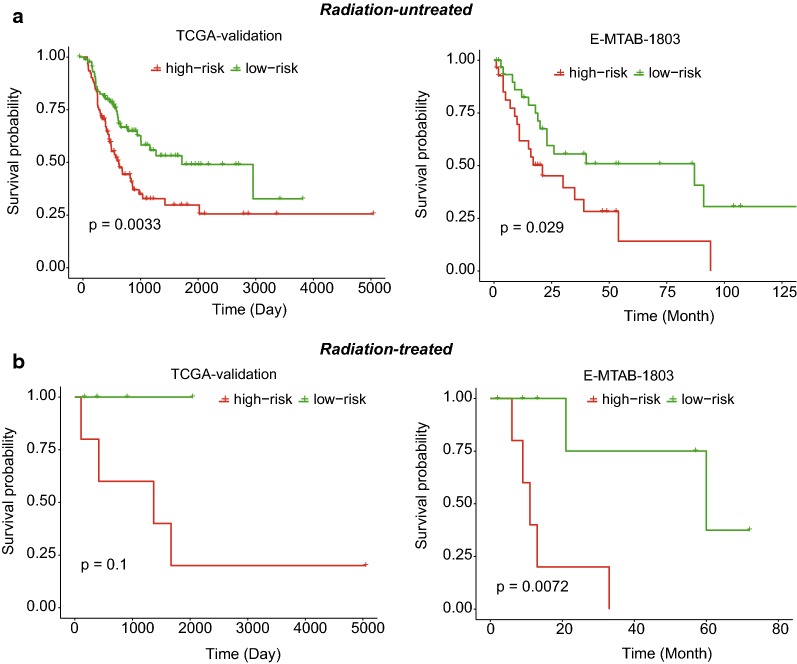
The independence of our stratification on the radiation treatment. The KM-curves for samples with and without radiation treatment were displayed in **b** and **a**

To further demonstrate that the stratification was a prognostic factor independent of clinically prognostic indicators and radiation treatment, we also conducted statistical tests on both TCGA-validation and E-MTAB-1803 datasets. Consistently, the high-risk and low-risk groups exhibited significant difference in overall survival among samples with or without lymph node metastasis, which were observed in both of the validation datasets (Fig. 3a, b, log-rank test, *P* < 0.05). Similarly, the high-risk group also exhibited worse overall survival than the low-risk group among samples with and without distant metastasis from TCGA-validation dataset, and among samples without distant metastasis from E-MTAB-1803 dataset (Fig. 3c, d, log-rank test, *P* < 0.05). Exceptionally, the statistical significance was not observed among samples with distant metastasis from E-MTAB-1803 dataset, which may be resulted from the small sample size (Fig. 3d, n = 29). Furthermore, the high-risk group had worse prognosis than the low-risk group among samples with and without radiation treatment from both TCGA-validation and E-MTAB-1803 dataset (Fig. 4, *P*-value < 0.1). These results demonstrated that the three-gene stratification of MIBC samples was a prognostic factor independent of both clinically prognostic indictors and radiation treatment.

### The biological differences between the two risk groups and potential therapeutic targets of the high-risk group

**Fig. 5:**
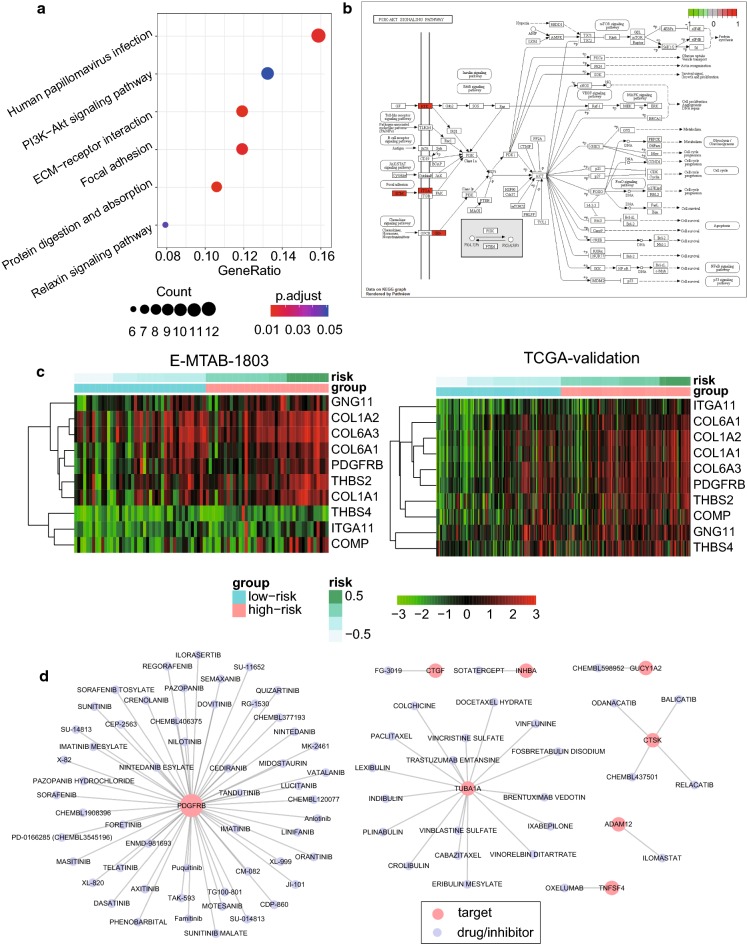
The dysregulated pathways in high-risk MIBC and potential targeted drugs. **a** The KEGG pathways enriched by the upregulated genes in high-risk MIBC. **b** The upregulated genes/components involved in PI3K-Akt signaling pathway. **c** The expression patterns of the genes involved in PI3K-Akt signaling pathway in the high-risk and low-risk groups. **d** The drug–target interaction network mapped by the upregulated genes. The red and purple nodes represent drug targets and drugs/inhibitors

To improve our understanding of the biological differences between these two risk groups, we performed differential gene expression analysis on the two validation datasets (t-test, adjusted P-value < 0.05). The upregulated and downregulated genes were subjected to KEGG enrichment analysis, respectively. However, the downregulated genes were not enriched in any KEGG pathways, while the upregulated genes were enriched in human papillomavirus infection, PI3K-Akt signaling pathway, ECM-receptor interaction, focal adhesion, protein digestion and absorption, and relaxin signaling pathway (Fig. 5a, FDR < 0.05). The co-occurrence of PI3K-Akt signaling pathway, ECM-receptor interaction, and focal adhesion suggested that these two risk groups showed significant difference in tumor microenvironment. Further investigation of PI3K-Akt signaling pathway highlighted the upregulated components, such as RTKs (receptor tyrosine kinases), ECM (extracellular matrix), and ITGA (Integrin alpha subunit) (Fig. 5b). The genes involved in PI3K-Akt signaling pathway were significantly upregulated in high-risk group as compared with the low-risk group (Fig. 5c).

To further search for drug and therapeutic targets for patients in the high-risk group, we mapped the upregulated genes in the high-risk group to the drug–target interaction network, and identified 8 genes, including *ADAM12* (ADAM Metallopeptidase Domain 12), *CTGF* (Cellular Communication Network Factor 2), *CTSK* (Cathepsin K), *GUCY1A2* (Guanylate Cyclase 1 Soluble Subunit Alpha 2), *INHBA* (Inhibin Subunit Beta A), *PDGFRB* (Platelet Derived Growth Factor Receptor Beta), *TNFSF4* (TNF Superfamily Member 4), and *TUBA1A* (Tubulin Alpha 1a), as the potential therapeutic targets (Fig. 5d). Notably, *PDGFRB*, a receptor for platelet-derived growth factor, was the most frequent target of several drugs, suggesting that the patients in high-risk group could be treated with the inhibitors of *PDGFRB*. In addition, *TUBA1A* was also identified as the potential target of multiple drugs for high-risk MIBC. Notably, a clinical trial was conducted to study the effectiveness of Ixabepilone, which was an inhibitor of *TUBA1A*, in treating patients with progressive or metastatic urinary tract cancer (The clinical trial accession: NCT00021099).

To prove the practicability of these target-drug/inhibitor predictions, we performed literature research for these pairs. Among the drugs targeting *PDGFRB* and *TUBA1A*, 6 and 4 drugs were reported to be used in the treatment of MIBC (Additional file 2: Table S2), respectively. Particularly, drugs of sorafenib, imatinib, dasatinib, sunitinib, vinflunine, vinblastine, trastuzumab emtansine, and trastuzumab for *PDGFRB* or *TUBA1A* were shown to have the potentials in treating MIBC by in vitro, in vivo or clinical trials. The mapping of the upregulated genes in high-risk group to drug–target interaction network provided multiple therapeutic candidates for the patients of high-risk group.

### The comparison of the prognostic stratification and TCGA classification

**Fig. 6:**
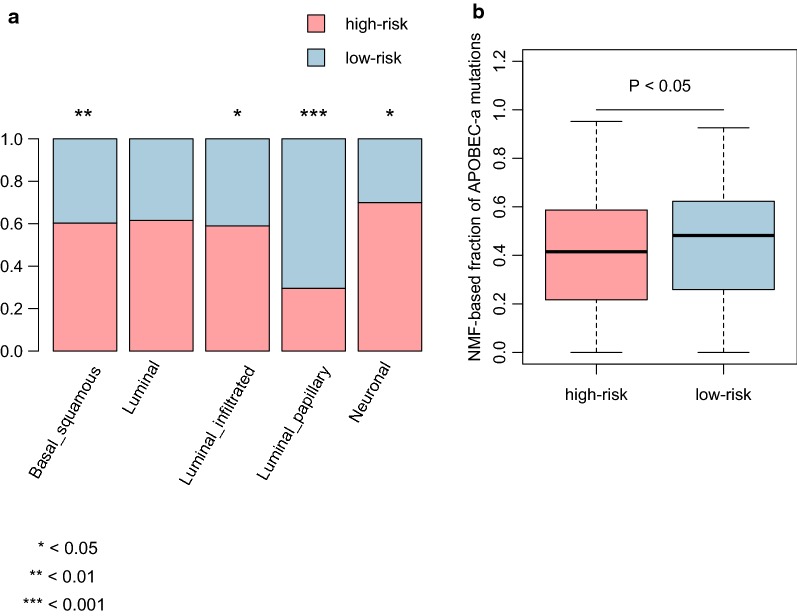
The comparison of the prognostic stratification with TCGA classification. **a** The proportion of high-risk and low-risk samples in each TCGA subtype. **b** NMF-based fraction of APOBEC-a mutations in high-risk and low-risk groups

We compared our prognostic stratification with the TCGA classification, and found that the Basal-squamous, Luminal infiltrated, and Neuronal subtypes were highly enriched in high-risk group (*P* < 0.05, Fig. 6a), In contrast, Luminal-papillary subtype was highly enriched in low-risk group (*P* < 0.05, Fig. 6a). For the five mutational signatures including, C>T_CpG, APOBEC-a, APOBEC-b, ERCC2, and POLE, only APOBEC-a was found to be higher in low-risk group than in high-risk group (*P* < 0.05, Fig. 6b), suggesting that the mutations of patients in low-risk group might be caused by the dysfunction of APOBEC3A. Furthermore, the results also suggested that the patients with APOBEC-a mutation signature might have a better prognosis.

### Identification of immune infiltration patterns for MIBC

**Fig. 7:**
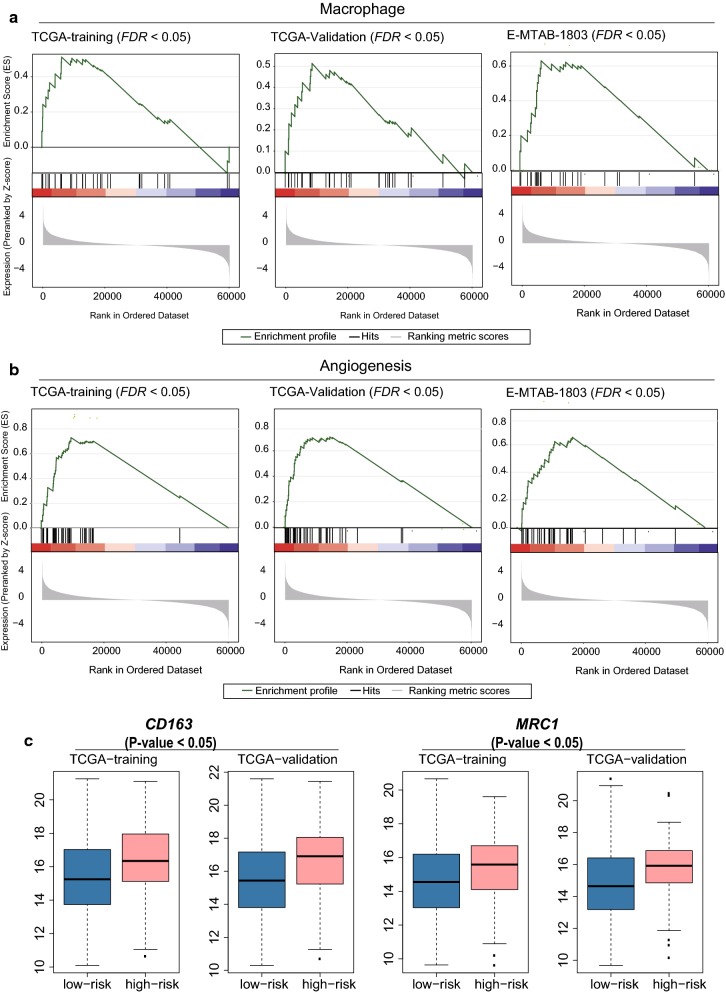
The infiltration of macrophage and hyperactivation of angiogenesis in high-risk MIBC. **a** The high infiltration of macrophage in high-risk MIBC by GSEA. **b** The heperactivation of angiogenesis in high-risk MIBC, which was revealed by the enrichment analysis of the angiogenesis-related genes. **c** The expression patterns of two M2 macrophage maker genes in TCGA-training and validation datasets

To reveal the landscape of immune cells infiltrating into tumor tissues of MIBC, we first collected 24 immune cell types and angiogenesis-related genes from the previous study [25]. The gene set enrichment analysis was performed to identify immune cells that exhibited more remarkable infiltration in high-risk MIBC as compared with low-risk samples. We found that macrophage was highly filtrated into tumors of the high-risk MIBC (Fig. 7a, FDR < 0.05). The angiogenesis-related genes were highly upregulated in high-risk MIBC (Fig. 7b, FDR < 0.05), suggesting that the angiogenesis was an important indicator for poor prognosis of MIBC. Moreover, two marker genes of M2 macrophage, *CD163* (CD163 molecule) and *MRC1* (Mannose Receptor C-Type 1), were observed significantly upregulated in high-risk MIBC (Fig. 7c, *P*-value < 0.05) in all datasets except M-TAB-1803 dataset due to the lack of probes quantifying related gene expressions. These results further suggested that M2 macrophage may be the major infiltrated immune cells in high-risk MIBC and promote the progression of MIBC.

## Discussion

Bladder cancer is one of the most common malignancies worldwide [1]. Several studies [8–10] have proposed several approaches to select and combine gene signatures for predicting the prognosis of MIBC, however, these gene signature sets have not been systematically compared with one another, and their performance on independent datasets are not satisfying. In the present study, we aimed to detect a combination of gene signatures for MIBC prognostic prediction and risk stratification. Based on the systematic data analysis, we identified three prognostic gene signatures, *KLK6*, *TNS1*, and *TRIM56*, as the best subset of genes. *KLK6*, a member of the kallikrein, was able to predict tumor recurrence in epithelial ovarian carcinoma [26]. Moreover, *KLK6* has been reported to regulate epithelial-to-mesenchymal transition (EMT) and serve as prognostic biomarker for head and neck squamous cell carcinoma patients [27], which also indicated that the poor prognosis in MIBC samples with high expression of *KLK6* might be associated with the dysfunction of EMT. *TNS1* was rarely reported to be associated with cancer, but was identified as a potential biomarker in human colorectal cancer [23] and a regulator of metastatic potential in colorectal cancer via altering expression of genes involved in cell motility [24]. In contrast, previous studies [28, 29] have identified *TRIM56* as a tumor suppressor through activation of TLR3/TRIF signaling pathway, which was consistent with the result that *TRIM56* expression was a favorable indicator of MIBC in this study. Utilizing the expression profiles of these three signatures, we successfully built a multivariable Cox regression model to calculate risk scores and stratified the MIBC patients into high and low risk groups.

To demonstrate the high performance of the prognostic stratification based on MIBC risk prediction, we selected two independent cohorts as validation datasets. Remarkably, the stratified groups in the two validation datasets both exhibited significant difference in overall survival (Fig. 2, *P* < 0.005). To further demonstrate the capability of the three-gene-signature in MIBC risk stratification, we also compared our three-gene-signature-based method with three other stratification methods by Wu et al. [10], Kim et al. [8, 9], and found that our method was superior to the others as patients stratified with our method exhibited a more significant difference in overall survival between high- and low-risk groups, suggesting that this prognostic stratification for MIBC was more robust and accurate. In addition, we also investigated whether this stratification was independent from other clinical indicators, such as lymph node and distant metastasis, and a history of radiation treatment, which could affect the MIBC prognosis. Consistently, the high-risk group exhibited worse prognosis than low-risk group in samples with and without lymph node metastasis, distant metastasis, and a history of radiation treatment. Specifically, we found that none of the three other stratifications selected the gene signatures based on univariable Cox analysis and their functionality. However, the present study selected the three gene signatures by integrating the univariable Cox analysis and Maximum Minimum Parents and Children (MMPC) algorithm, the strength of which is the maintenance of the statistical significance in both univariable and multivariable analyses, not only in univariable analysis.

Moreover, PI3K-Akt signaling pathway, a critical signaling pathway for cancer cell formation and progression [30–33], was highly activated in the high-risk group according to the results from differential expression analysis and gene set enrichment analysis. In addition to PDGFRB, other upstream receptor tyrosine-kinases (RTKs) in PI3K-Akt signaling pathway, such as *EGFR*, *CSF1R*, *FGFR1*, *FLT4*, *FLT3*, *NGFR*, *NTRK1*, *PDGFRA*, and *TEK*, were also observed to be upregulated in the high-risk group (P < 0.05, Additional file 3: Figure S1). These results further suggested that overexpression of these RTKs may be responsible for PI3K-Akt signaling pathway hyper-activation, and RTKs may serve as therapeutic targets in high-risk MIBC. Recently, an FGFR family inhibitor, erdafitinib, was approved by the U.S. Food and Drug Administration (FDA) to treat locally advanced or metastatic bladder cancer in adult patients with susceptible genetic alteration in *FGFR3* or *FGFR2*, whose condition still progressed during or following prior platinum-containing chemotherapy. Therefore, we proposed that the erdafitinib treatment may work on patients of high-risk group, when platinum-containing chemotherapy failed to bring satisfying results.

In general, immune cells were infiltrated into tumor cells. We found that macrophage was highly filtrated into the high-risk MIBC (Fig. 7a, FDR < 0.05), and the angiogenesis-related genes were highly upregulated in high-risk MIBC (Fig. 7b, FDR < 0.05). More importantly, two M2 macrophage markers, CD163 and MRC1, were observed to be significantly upregulated in high-risk MIBC (Fig. 7c, P-value < 0.05). The co-occurrence of M2 macrophage infiltration and hyper-active angiogenesis in high-risk samples suggested that M2 macrophage may promote the angiogenesis of high-risk MIBC, which was consistent with previous studies [34–36].

However, the present study still has some limitations. First, gene expression profiles of patients with long-term follow-ups should be collected to further assess the robustness of our stratification. Second, data regarding the efficacy of certain drugs in high-risk MIBC are not available, and in vitro and in vivo studies are needed to yield more experimental evidences. There is no experiment to validate the association between M2 macrophage and angiogenesis. Nevertherless, this study provides a new perspective on the molecular mechanisms behind high-risk MIBC, and has successfully illustrated how these mechanisms are related to the prognostic outcomes of MIBC patients.

## Conclusions

The present study has investigated the underlying molecular mechanism and potential therapeutic targets associated with worse prognosis of high-risk MIBC, which could improve our understanding of the progression of MIBC and provide new therapeutic targets for the management of MIBC.

## Supplementary information

**Additional file 1: Table S1.** The predicted pathways that the signature genes may participate in.

**Additional file 2: Table S2.** The potential anticancer drugs for MIBC based on the literature mining.

**Additional file 3: Figure S1.** The differential expression significance of Receptor Tyrosine Kinases (RTKs) between high-risk and low-risk groups.

## Data Availability

The datasets used and/or analyzed during the current study are available from the corresponding author on reasonable request.

## Abbreviations

MIBC: muscle-invasive bladder cancer
NMIBC: non-muscle-invasive bladder cancer
GWAS: genome-wide association studies
lncRNA: long non-coding RNAs
MMPC: maximum minimum parents and children
DGIdb: Drug Gene Interaction Database
RTKs: receptor tyrosine kinases
ECM: extracellular matrix
ITGA: integrin alpha subunit
