# Supplementary figures and images for: Systematic analysis of gene expression profiles reveals prognostic stratification and underlying mechanisms for muscle-invasive bladder cancer
Source: Cancer Cell Int. 2019 Dec 16;19:337. doi: 10.1186/s12935-019-1056-y (PMC6916460; doi:10.1186/s12935-019-1056-y)

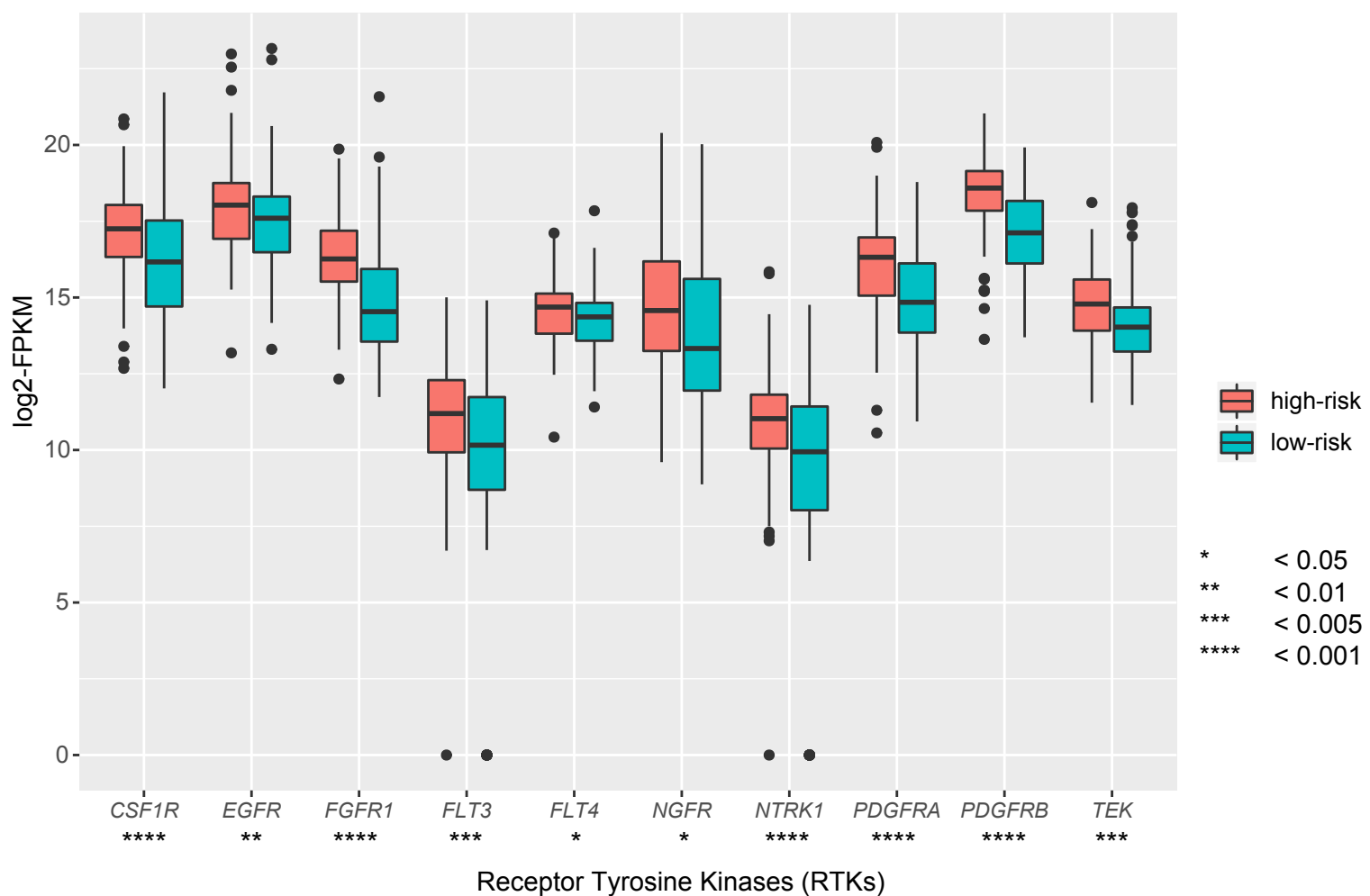

Supplement: Supplementary file 3 — Additional file 3: Figure S1. The differential expression significance of Receptor Tyrosine Kinases (RTKs) between high-risk and low-risk groups. [file 12935_2019_1056_MOESM3_ESM.pdf]
